# Short report: moderations in exercise motivation – gender and age moderates the relations of motivation quality and exercise behavior

**DOI:** 10.1080/21642850.2018.1462706

**Published:** 2018-04-18

**Authors:** Karin Weman Josefsson, Urban Johnson, Magnus Lindwall

**Affiliations:** a Research on Welfare, Health and Sport, Halmstad University, Halmstad, Sweden; b Department of Psychology, University of Gothenburg, Gothenburg, Sweden

**Keywords:** Exercise, moderation, motivation, self-determination theory

## Abstract

**Aims:**

Self-determined motivation has been found to be an important predictor of exercise behavior. Findings on gender and age differences are however mixed and previous research has called for studies to examine gender and age as potential moderating factors as they might influence how motivation quality affects exercise behavior.

**Methods:**

Embedded in a controlled trial of a digital intervention aiming to promote exercise motivation, this study examined specific (longitudinal) pathways related to motivation quality, psychological need satisfaction and exercise behavior within the self-determination theory (SDT) process model in a sample of 318 adult employees. The participants completed web-based versions of Basic Psychological Needs in Exercise Scale, Behavioural Regulations in Exercise Questionnaire-2, and Leisure Time Exercise Questionnaire three times during a six weeks period.

**Results:**

Moderation analyses revealed significant gender and age differences in the associations of motivation quality, basic psychological needs and exercise behavior over time. Several paths in the SDT-process model, linking psychological needs and motivation quality to exercise behavior, were moderated by gender and age. The stipulated mechanisms between exercise, motivation and psychological need satisfaction in the SDT-process model revealed to be stronger for women than for men, and stronger for older adults than for younger and middle-aged adults. The effect of amotivation on exercise was also significantly moderated by age in the full sample, by positively predicting light exercise for younger adults.

**Conclusions:**

Future recommendations are related to the examination of potential differences in opportunities of autonomy support in the social context based on factors such as gender and age, and also to further examine these factors as potential moderators instead of statistically controlling them as default.

## Introduction

**Figure 1.**
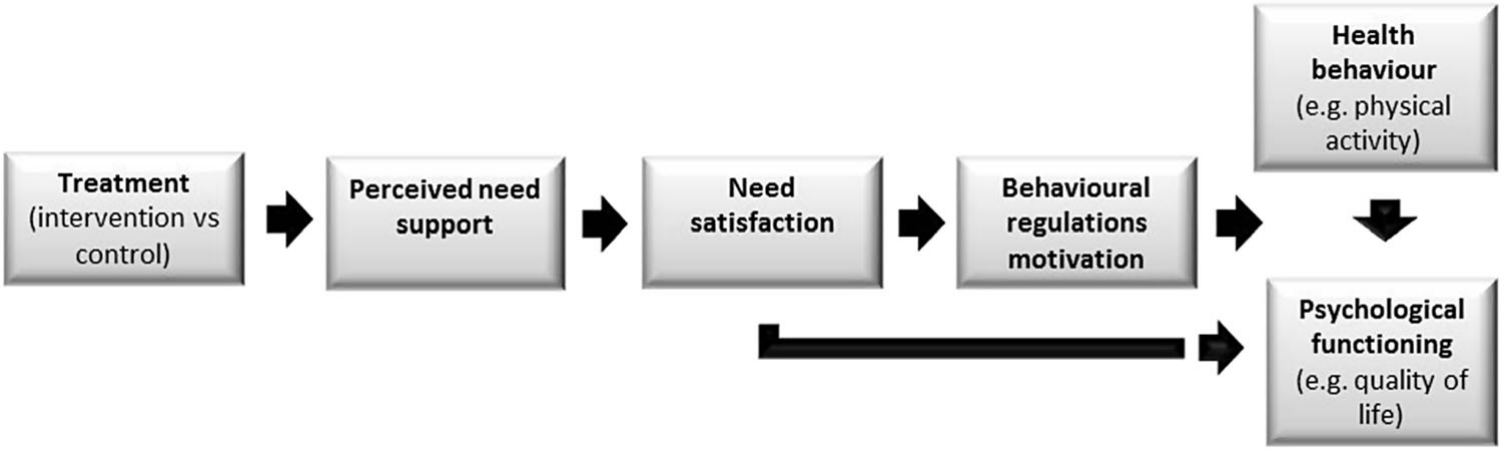
SDT-process model. From Fortier et al., 2012, p. 3, with permission.

This short report describes a study that is part of a published controlled trial (see Weman Josefsson, Johnson, & Lindwall, 2016), building on the previous calls for further exploration of the potential moderating effects of gender and age within the field of exercise motivation (Fortier, Duda, Guérin, & Teixeira, 2012; Guérin, Bales, Fortier, & Sweet, 2012; Owen, Smith, & Lubans, 2014; Teixeira, Carraca, Markland, Silva, & Ryan, 2012). Previous findings on gender and age differences are mixed (Weman-Josefsson, Lindwall, & Ivarsson, 2015) and it would be an interesting avenue to extend current knowledge by examining how these potential moderators might influence the effect of motivation quality on exercise. Such knowledge may inform the application of uniform or segregated tailoring of exercise interventions. Self-determination theory (SDT; Deci & Ryan, 1985; 2000) highlights the importance of motivation quality for sustainable adoption of health behaviors such as exercise. Motivation quality is presented on a continuum stretching from amotivation (non-regulation) to intrinsic (or self-determined) motivation with four types of external behavioral regulations with varying degrees of self-determination in between. External regulation is about behavior driven by rewards or punishment, while introjects is about pride or shame. Identified regulation is about activities that provide values, meaning and personal relevance and integrated regulation is a highly internalized form of regulation connected to identity and beliefs in line with life goals. External and introjected regulations represent controlled types of motivation while identified and integrated regulations represent more self-determined types of motivation. Intrinsic motivation is the most self-determined form, when we do things for joy and satisfaction in the activity itself. It is suggested that when certain basic psychological needs for competence (feeling capable), autonomy (feelings of choice and volition) and relatedness (feeling connected with others) are satisfied in an exercise context, the person is expected to internalize reasons to engage in the behavior and controlled motivations will thereby be internalized into more self-determined motivation. In the application of SDT tenets, practitioners use ‘autonomy support’, whereby social contexts (and interactions) can be modified to satisfy the three basic psychological needs and facilitate internalization and sustained behaviors (Deci & Ryan, 2000). The relations between psychological need satisfaction, motivation quality and behavior are described in a process model (see Figure 1).

The sequence linking psychological needs and motivation to behavior is thought to be universal (i.e. not differ) across genders, ages and cultures (Deci & Ryan, 2000), but the findings vary and many cross-study comparisons (especially for gender) are inconsistent. A possible explanation is that factors such as gender and age might affect not only fulfillment of the needs and type of motivation in the context of physical activity and exercise – which has been explored in previous research using typical comparisons (i.e. simpler analyses such as correlations and ANOVA’s) – but also the internalization process and the pathways linking motivation type, need satisfaction and behavior together (see Weman-Josefsson et al., 2015). Analyzing pathways and processes, however, demand other types of more sophisticated analytical approaches, enabling moderation and mediation analyses (Hayes, 2013) which might shed light on the universality of motivational mechanisms. Levels of physical activity in childhood and adolescence have been found to predict levels of physical activity in adulthood (Telama et al., 2005), and it is likely that goals and values behind these behaviors change over the course of a lifetime (Brunet & Sabiston, 2011), and that different forms of motivation (i.e. motivation quality) are more prominent at different stages of life (Owen et al., 2014). For example, external motivation for physical activity seems to become less important as people age (Beck, Gillison, & Standage, 2010), and accordingly external motivation was a significant predictor for physical activity in adults aged 18–24 but not adults aged 25–64 as reported in the study by Brunet and Sabiston (2011).

Weman-Josefsson et al. (2015) found age to moderate associations between motivational regulations (i.e. motivation quality) and exercise behavior. Moderation analyses showed intrinsic regulation to positively predict exercise for the older group of adults (46–78 years), but not for the younger group (18–45 years), whereas identified regulation was a stronger exercise predictor among the younger group (Weman-Josefsson et al., 2015). Intrinsic regulation predicting exercise is in line with the previous research mentioned above (see Beck et al., 2010; Brunet & Sabiston, 2011), but we still seem to know little about physical activity motivation in adulthood. In line with suggestions of universality within the SDT framework (Deci & Ryan, 2000; Ryan & Deci, 2002), a meta-analysis by Guérin et al. (2012) concluded that differences in behavioral regulations between men and women are trivial in exercise contexts. Exercise motivation has, on the other hand, been found to differ between women and men (e.g. Hamilton, Cox, & White, 2012; Li, 1999), and Weman-Josefsson et al. (2015) found gender to moderate the relations between motivation and exercise behavior. Autonomous motivation (especially identified regulation) was found to be a stronger predictor of exercise for women, whereas controlled motivation (external and introjected) was positively associated with exercise in men, but not in women (Weman-Josefsson et al., 2015). These results are inconsistent compared with the findings of Guérin et al. (2012), but notably their meta-analysis did not consider the different paths from motivational source to outcome (i.e. the mediating mechanisms of the process model) as Weman-Josefsson et al. (2015) did. The question of whether applying uniform (one-size-fits-all) or segregated (personalized) tailoring of exercise and physical activity interventions based on age and gender differences remains mainly unexamined and deeper exploration of this issue may spawn valuable knowledge for theory and practice.

Embedded in a controlled trial of a digital intervention aiming to promote exercise motivation, this study will examine the specific pathways specified in the SDT-process model related to motivation quality, psychological need satisfaction and exercise behavior. The main study showed no moderating effects of gender and age in the effect of the intervention which might indicate that the intervention had general effects across gender and age groups. To follow up with previous cross-sectional studies showing gender and age effects (mediation and moderation) in a similar sample, we wanted to study cross-sectional analyses of gender and age for each time-point and between time-points to see if these findings were repeated. The main focus of this study is, therefore, to examine if gender and age operate as moderators in the relations between psychological need satisfaction; motivation quality and exercise behavior within the SDT-process model.

## Methods

### Study design and participants

This study was embedded in a digital intervention aiming to promote exercise motivation (see detailed description in Weman-Josefsson, Johnson, & Lindwall, 2016) targeting a sample of 318 adult women (*n* = 278) and men (*n* = 40) aged 23–67 years (*M* = 46.7; SD = 9.4) who participated in a step contest in a digital step contest provided by their employer. Most companies had arranged this contest several times before and their employees could choose to compete individually or in teams (or not to join the event). The competition is usually framed as a social, health-related work-place event. The employees who signed up for the step contest were subsequently invited to participate in this study. All study participants provided informed consent. In the main study, participants were stratified by age and gender and assigned to either the control (*n* = 152) or experimental (*n* = 166) group, all of which is described in a CONSORT protocol and flow-chart published together with the main article. The intervention was delivered via a web-based application, adaptable to tablet/smartphone. From an SDT perspective, the underlying intention of the digital intervention was to influence participants’ exercise behaviors by manipulating the suggested causal mechanisms described in the process model, that is, through facilitating internalization by providing digital autonomy support, structure and involvement. It is important to note that this short report does not concern experimental/control conditions or intervention effects; this paper is about analyses of the full sample. All participants completed validated web-based versions of the following instruments: Basic Psychological Needs in Exercise Scale (BPNES; Vlachopoulos & Michailidou, 2006), Behavioural Regulations in Exercise Questionnaire-2 (BREQ-2; Markland & Tobin, 2004) and Leisure Time Exercise Questionnaire (LTEQ; Godin & Shephard, 1985; Godin & Shephard, 1997). LTEQ provides self-reported scores on light, moderate and strenuous exercise, and these three scores can be calculated into a total exercise score of metabolic equivalent of exercise (MET). To perform analyses based on age, the participants were divided into three age groups representing younger adults 23–34 years (26 women; 7 men), middle-aged adults 35–54 years (174 women; 24 men) and older adults 55–67 years (66 women; 9men). Cronbach’s alpha for the BPNES ranged from .84 to .93 and for BREQ-2 from .71 to .75. Motivation quality, psychological need satisfaction and exercise behavior were measured using a three-wave web-based questionnaire (T1 = baseline; T2 = week 3 and T3 = week 6; see Tables 1 and 2). Following the step contest, intervention took place between T1 and T2, with T3 as follow-up. Of the 318 adults included in the study, 187 participated in all three measurement points and drop-out analyses (*t*-tests) showed that participants with high amotivation levels at T2 were more likely to drop out from the study.

**Table 1.** Methodological characteristics (content) of the intervention trial.

Description	Delivery/assessment
Main SDT components	*Autonomy*: motivational readiness/stage based support, offer choice/options/decisions, exploration of exercise goals and congruence of values/life goals, promote sense of ownership and self-initiation, avoid controlling language
	*Relatedness*: provide involvement, failure normalization, functions for sharing, role model stories
	*Competence*: provide structure, goal setting support, exercise-barrier identification and relapse prevention, self-regulation/self-monitoring, exercise related health literacy and relevant web services/links
SDT outcomes & measures	Perceptions of psychological needs satisfaction in exercise (BPNES) and behavioral regulations in exercise 2 (BREQ-2). Cronbach’s alpha for the BPNES ranged from .84 to .93 and for BREQ-2 from .71 to .75
Behavioral outcomes	Self-reported exercise behavior (LTEQ).
Assessment time-points	Experimental group and control group collectively: (a) initial informed consent providing demographics for randomization, (b) baseline T1, (c) post-intervention T2 and (d) follow-up T3

**Table 2.** Overview of main study design, results and contributions.

	Description
Design	Three-wave controlled trial intervention
Theoretical foundation	Self-determination theory
Participants	318 adult women (*n *= 278) and men (*n *= 40) aged 23–67 years (*M *= 46.7; SD* *= 9.4) participating in a step contest provided by their employer
Measures	BPNES, BREQ-2, LTEQ
Analyses	ANCOVA and MVA with bootstrapping resampling approach and asymmetric 95% confidence interval based on 5000 resamples
Results	The intervention increased levels of total, strenuous and light exercise and predicted mediators in terms of motivational quality. The intervention decreased controlled motivation (external regulation) and amotivation Amotivation was involved in statistically significant main (time) effects, as well as in mediating the intervention effects (the decrease in amotivation mediated the levels of light exercise post-intervention)
Strengths and Limitations	Important strengths are the controlled design, the use of advanced moderation analyses, the sample of middle-aged non-clinical adults and the three-wave measurements. No unexpected disparities were found in the *t*-test drop-out analysis, yet drop-out might have influenced power and analysis precision. In terms of limitations, the use of self-reports, relatively high mean age, and uneven distribution of men and women in this sample might have impacted the results.
Conclusions	The findings indicate gender and age differences at a higher level (in terms of moderators, not mean levels) and highlight the need for future studies to address these relationships more thoroughly than usually done.

All moderation analyses were performed using the SPSS macro PROCESS, as recommended by Hayes (2013) and a bootstrapping resampling approach was used to calculate product-of-coefficients and an asymmetric 95% confidence interval based on 5000 resamples (Preacher & Hayes, 2004; Preacher & Hayes, 2008).

### Ethics statement

The intervention trial was approved by the regional ethics board (Dnr. Etik 2014/336) and guided by the CONSORT checklist (see supplemental material in the main article by Weman-Josefsson et al., 2016). Participation was voluntary and all respondents completed informed consent.

## Results

The main effects of the intervention on psychological need satisfaction, motivation quality and exercise are described in the paper by Weman-Josefsson et al. (2016). There were no moderating effects of gender and age in the effect of the intervention, but gender and age moderated the specific effects of the associations between exercise and motivation in the full sample.

### Moderation effects of gender

The path between external motivation at T1 and total exercise T1 was negative and significant for women (*β* = −7.19, *p* < .05), but positive and non-significant for men. Similarly, external regulation T2 predicted total exercise at T3 for men in a positive direction (*β* = 11.29, *p* < .01), while it was negative and non-significant for women. Identified regulation at T2 had a negative significant relation to strenuous exercise at the same time-point for men (*β* = −16.52, *p* < .01), but had a positive and non-significant relation in women. Except for identified regulation (*r*^2^ = .13), explained variances for the whole group were small (*r*^2^ = .03). Gender was also found to moderate the relationships between motivational regulations and exercise as well as between motivational regulations and psychological need satisfaction. Intrinsic regulation at T2 positively predicted relatedness need satisfaction for women at the same time-point (*β* = 0.47, *p* < .05), but this path was negative and non-significant for men. Additionally, relatedness need satisfaction at T2 was positively associated with identified regulation at the same time-point in women (*β* = 0.37, *p* < .05), but was not related to motivation in men. Relatedness need satisfaction at T2 had a negative relation to external regulation at the same time-point in women (*β* = −0.13, *p* < .05), but not in men. Finally, intrinsic motivation at T2 was more strongly related to autonomy need satisfaction at T3 for women (*β* = 0.78, *p* < .05), than for men (*β* = 0.28, *p* < .05).

### Moderating effects of age

A negative association between external regulation T2 and strenuous exercise at T3 was stronger and significant for older adults (*β* = −8.90, *p* < .01) compared to middle-aged adults and was positive (but non-significant) for younger adults. The paths between amotivation at T1 and light exercise at T1 (*β* = 3.59, *p* < .05) and at T2 (*β* = 3.45, *p* < .05) were positive and significant for younger adults, but weaker and non-significant for middle-aged adults and negative and non-significant for older adults. Similarly, the effect of amotivation at T1 on light exercise at T2 was positive and significant for young adults (*β* = 3.45, *p* < .05), but weaker and non-significant for middle-aged adults and negative and non-significant for older adults. Additionally, there was a significant and positive path between autonomy at T3 and moderate exercise at T3 and it was stronger for older (*β* = 4.38, *p* < .05) and middle-aged adults (*β* = 1.41, *p* < .05) than for younger adults. Competence need satisfaction (*β* = 4.13, *p* < .05) and global need satisfaction (*β* = 1.50, *p* < .05) at T3 positively predicted moderate exercise at that time-point for older adults, but not for middle-aged adults and these paths were negative and non-significant for younger adults. Explained variance ranged from *r*^2^ .03 to .07.

## Discussion

The purpose of this study was to examine potential moderation effects of gender and age in the mechanisms of the SDT-process model. Overall, the general trend in the current analyses was that most effects did not differ between subgroups, but moderating effects of both gender and age were found, also over time. In other words, we found gender and age to moderate some of the relations between motivation quality, psychological need satisfaction and exercise behavior.

The findings regarding gender are consistent with the cross-sectional results found by Weman-Josefsson et al. (2015) in a digital context, which also showed external regulations predicting exercise for men, whereas identified regulation predicted exercise for women. Especially interesting are the reversed paths for different groups: for example, identified regulation had a negative relationship with exercise solely for men, and extrinsic regulation had a negative relationship with exercise just for women – and vice versa. If these relationships can be verified in future studies and in various contexts, it will not only carry implications for practice but may also bring forward a theoretical discussion about the proposed universality of the paths in the SDT-process model. Overall, the patterns displayed above imply that the proposed mechanisms between exercise, motivation and psychological need satisfaction (see e.g. Fortier et al., 2012) in this study hold for women, but not for men. The competition context of the intervention study could be part of the explanation, and maybe men are more regulated by the external rewards (i.e. winning) than women, who perhaps participate for more autonomous and social reasons. This is a suggestion reinforced by previous studies indicating that women tend to be more engaged in internet-based physical activity programs and interventions than men (Brouwer et al., 2010; Dawson, Tracey, & Berry, 2008; Napolitano et al., 2003). The finding that men seems more driven by controlled motivation is in line with the suggestion by Deci and Ryan (1985) that these regulations might work in the short term (being a study spanning only six weeks), in combination with men plausibly joining this work-site based competition for extrinsic reasons such as winning, or for work-group induced pressures. Knowledge of how to tailor such digital services to tap autonomy supportive structures based on what works for women and men respectively might benefit the tailoring of digital services and programs to fit their preferences and thereby facilitate maintenance and reduce potential drop-out rates. It is important to note that the step contest was set up by the employers, and was not part of the intervention; the participants were invited to this study in conjunction with that event. This is a quite interesting yet challenging setup, and in the main study, we suggest that the digital intervention might buffer from the potential negative effects of the competition, perhaps differently for different subgroups. For example, the push and pull of competitions might work differently for men and women (at least in digital settings where our previous studies have shown women to have more self-determined types of motivation to participate) and framing aspects such as goal setting or behavior rationale in different ways might affect need satisfaction and internalization differently for men and women.

Different forms of motivation are likely to vary at different stages of life (Brunet & Sabiston, 2011; Owen et al., 2014). On a general level, the results for middle-aged and older adults in the current study are in line with theoretical expectations, but the inverted paths for younger adults are more difficult to explain from an SDT point of view, and might have underpinnings in the social environmental context of this study. Even though the results are similar to the moderations of age found in Weman-Josefsson et al’s. (2015) study, a further examination of these connections, preferably also testing mediated moderation and/or moderated mediation (Fortier et al., 2011), could shed more light on the actual nature of such links in different age groups. Probably the most intriguing discovery in moderations of age was that the paths between amotivation at baseline and light exercise post-intervention were positive and significant for young adults. This link is quite surprising and has to our knowledge not been observed before, not even in the extensive review by Teixeira and colleagues (2012). The reasons behind this could be due to the unlikeliness in itself with regard to theoretical explanations (Hardcastle et al., 2015; Teixeira et al., 2012). That is because amotivation denotes the absence of motivation representing a complete lack of self-determination (Deci & Ryan, 2000) amotivated people are therefore unlikely to invest effort in (Hardcastle et al., 2015), or carry intentions towards (Thøgersen-Ntoumani & Ntoumanis, 2006) health behaviors. Thus, they would be considered improbable to spot in exercise contexts (Teixeira et al., 2012). Nevertheless, it is possible to (passively) participate in exercise activities in spite of experiencing amotivation, which means they may participate without true intention (Deci & Ryan, 2000). Amotivated individuals could, therefore, engage in exercise, but without really knowing why they should do it. Perhaps these young adults also experience amotivation because they do not find light exercise meaningful. Nevertheless, in the main study results showed that higher levels of amotivation at baseline were associated with more exercise post-intervention in the younger age group.

Referring to the discussion above, maybe the amotivated participants experienced non-volition due to work-site related group pressures. For example, they might have felt socially pressured to join a team of colleagues or (indirectly) pressured in general to participate in activities organized by the employer.

Even if only preliminary conclusions can be drawn here, the findings provide indications for gender and age differences at a higher level (i.e. in terms of associations/paths and not just mean levels) and highlight the need for future studies to address these possible relationships specifically for different subgroups. The present results imply that gender and age might act as moderators buffering potential adverse (psychological need thwarting) effects of competitive settings for certain subgroups, in this case, women and older adults. Few studies have examined these paths before, and the moderation results reported here together with our previous study (Weman-Josefsson et al., 2015) indicate effects of gender and age should not be ignored or statistically adjusting for as usually done. A possible theoretical explanation to the current findings might be that the moderations of gender and age are a result of how the social context contributes differently to need satisfaction (or need frustration) for these subgroups. In that case, the results might not contradict the universality contention of SDT but offer a better understanding of intervention tailoring, suggesting that more segregated applications could be a fruitful approach. All in all, this short report sheds some light on potential subgroup differences and future interventions that could do well in addressing moderating effects to tailor differently for subgroups based on gender and age.

### Limitations

Important strengths of the study would be the use of advanced moderation analyses, the relatively large sample of middle-aged non-clinical adults and the three-wave measurements. No unexpected disparities were found in the *t*-test drop-out analysis, yet drop-out might have influenced power and analysis precision. In terms of limitations, the use of self-reports is an apparent weakness as in many similar studies. Also, the use of a combined sample of control and experimental groups might be viewed as controversial, but as pointed out above, we were interested in the overall mechanisms of motivation and felt this sample would make a feasible opportunity to study these factors, also considering the lack of previous works. The specific context of a digital step contest and the relatively high mean age, along with the uneven distribution of men and women in this sample might have impacted the results. Yet, the distribution of gender reflects a common situation, because women seem to be more prone to join web-based PA and exercise interventions (see e.g. Brouwer et al., 2010; Dawson et al., 2008). The specificity of the sample, setting and context (digital setting, participants in a step contest at work) makes the findings hard to generalize to other contexts. We encourage more moderation studies across settings to clear the fog on how these mechanisms might work.

## Conclusions

The results of the present study suggest that motivational mechanisms can vary depending on subgroups based on gender and age. This adds to the knowledge on exercise motivation in a digital context and might translate into more specific tailoring of the digital intervention as well as the web-based step contest based on age and gender preferences as mentioned above. Perhaps also intervention tailoring could benefit from taking specific contextual influences (such as autonomy support) that are likely to modify vital prerequisites of certain subgroups into account. Future studies of how social contexts might differ in terms of autonomy support or opportunities of barrier management might contribute to increased understanding of the mechanisms found in this study between men and women or for different age groups. The significance of social context is clearly stressed within SDT (Deci & Ryan, 1985; Deci & Ryan, 2000), and potential discrepancies across genders, ages or cultures due to contextual factors would therefore not necessarily conflict with SDT principles (Fortier et al., 2012; Weman-Josefsson et al., 2015; Weman Josefsson, 2016). Deeper knowledge on such (social) factors would benefit further development of exercise and physical activity interventions. The purpose of this short report was mainly to explore the presence of potential mechanisms, and even if preliminary interpretations are made here, future studies are recommended to further examine the moderating effects of gender and age to provide elaborate and sensible explanations.
